# Savings in visuomotor learning are associated with connectivity changes within a cerebello-thalamo-cortical network encoding movement errors

**DOI:** 10.1007/s00429-025-03013-4

**Published:** 2025-10-13

**Authors:** Lucas Struber, Laurent Lamalle, Pierre-Alain Barraud, Aurélien Courvoisier, Rafael Laboissière, Takayuki Ito, Vincent Nougier, David J Ostry, Fabien Cignetti

**Affiliations:** 1 Univ. Grenoble Alpes, CNRS, UMR 5525, VetAgro Sup, Grenoble INP, TIMC, 38000 Grenoble, France; 2 Univ. Grenoble Alpes, Inserm US17, CNRS UAR 3552, CHU Grenoble Alpes, IRMaGe, Grenoble, France; 3 Grenoble Alps Scoliosis and Spine Center, Grenoble Alps University Hospital, Bvd de la Chantourne, CEDEX 09, 38043 Grenoble, France; 4 Univ. Grenoble Alpes, CNRS, LPNC, 38000 Grenoble, France; 5 Univ. Grenoble Alpes, CNRS, Grenoble-INP, GIPSA-Lab, 38000 Grenoble, France; 6 Department of Psychology, McGill University, Montreal, QC H3A1G1 Canada; 7 Yale Child Study Center, Yale School of Medicine, New Haven, CT 06511 USA; 8 Univ. Grenoble Alpes, Inserm, U1216, Grenoble Institut Neurosciences, 38000 Grenoble, France

**Keywords:** Motor learning, Movement error, Savings, fMRI, Functional connectivity

## Abstract

Savings refer to faster relearning upon re-exposure to a previously experienced movement perturbation. One theory posits that the brain recognizes past errors, enabling more efficient learning from them. If this is the case, there should be a modification in the neural response to errors during re-exposure to the perturbation. To investigate this hypothesis, we used fMRI to measure brain activity as participants adapted to a visuomotor perturbation across two sessions spaced one day apart, focusing on neural responses to movement errors. The magnitude of the movement error was incorporated into different types of GLMs to study error-related activation and co-activation (or functional connectivity). We identified a cerebello-thalamo-cortical network involved in processing movement errors during adaptation. We observed strengthened connectivity within this network during re-adaptation, particularly between the cerebellar lobule VI and the ventrolateral thalamus, as well as between the primary somatosensory cortex and the rostral cingulate motor zone. Importantly, participants with the greatest increases in connectivity strength also exhibited the largest amounts of savings. These results establish a link between the brain’s ability to represent errors and the phenomenon of savings.

**Supplementary Information:**

The online version contains supplementary material available at 10.1007/s00429-025-03013-4.

## Introduction

The formation of motor memory has traditionally been studied within adaptation paradigms, where a perturbation is introduced to disrupt a well-practiced behavior, such as point-to-point reaching. Initially, the imposed perturbation leads to significant movement errors; however, performance typically returns to near baseline levels within tens of trials (Haith and Krakauer 2013; Krakauer 2009). Classic examples of this include the adaptation of reaching movements when visual feedback is rotated on a virtual display (Krakauer 2009; Krakauer et al. 2005) or the introduction of force fields that alter the arm’s dynamics (Shadmehr and Brashers-Krug 1997; Shadmehr and Mussa-Ivaldi 1994). A key phenomenon observed in these adaptation paradigms is savings, wherein error reduction occurs more rapidly (i.e., in fewer trials) upon re-exposure to a previously experienced perturbation (Kojima et al. 2004; Krakauer et al. 2005; Smith et al. 2006; Zarahn et al. 2008). This is considered a manifestation of motor memory, which may result either from the accelerated recall of reinforced actions (Huang et al. 2011) or from increased sensitivity to past errors (Herzfeld et al. 2014). What remains unclear, however, is the precise brain changes that underlie savings.

At the brain level, it is generally believed that memories formed during motor adaptation are stored in cortical motor and somatosensory regions (Ebrahimi and Ostry 2024; Galea et al. 2011; Hadipour-Niktarash et al. 2007; Landi et al. 2011). However, the connection between these regions and faster relearning remains unclear. Modulation of excitability in these regions using transcranial magnetic stimulation has sometimes been shown to influence savings (Kumar et al. 2019; Villalta et al. 2015), though not always (Darainy et al. 2023). Similarly, there is no consensus among functional neuroimaging studies regarding the specific brain regions and mechanisms involved in savings. Some studies have failed to find any relationship between brain activity and savings (Bédard and Sanes 2014; Della-Maggiore et al. 2017), while others have reported associations, primarily with non-sensorimotor regions such as the hippocampus and areas of the default-mode network (Cassady et al. 2018; Gale et al. 2022; Standage et al. 2023). However, changes in the neuronal population activity dynamics in the primary motor cortex of monkeys have been shown to correlate with faster relearning (Sun et al. 2022). Furthermore, evidence suggests that the cerebellum may also contribute to the retention of motor memory (Herzfeld et al. 2014), including savings observed during relearning (Medina et al. 2001). An fMRI study even demonstrated increased activity in the cerebellum (lobule VI) when repeating a motor adaptation task, with this activity correlating with the amount of savings (Debas et al. 2010). Lastly, in locomotor adaptation, a relationship was identified between cerebellar-thalamic intrinsic functional connectivity and faster relearning (Mawase et al. 2017). These findings suggest that savings may be supported by neural changes occurring within a large-scale cerebello-thalamo-cortical network.

In this study, we assessed the neural basis of savings by investigating whether this phenomenon specifically relies on brain areas involved in coding movement errors. It has long been established that the brain is attuned to errors, with activation, particularly in sensorimotor cortical and cerebellar regions, as well as parietal association areas, being correlated with errors during motor performance (Diedrichsen et al. 2005; Grafton et al. 2008; Luauté et al. 2009). Moreover, changes in intrinsic functional connectivity after learning are primarily observed in error-related regions (Bernardi et al., 2018), suggesting that these areas are crucial for motor memory formation. Based on these findings, we hypothesized that improvements in learning, reflected in savings, result from changes in these error-related brain regions. Specifically, we proposed that these changes would be evident in the activation and coactivation (i.e., functional connectivity) of these areas. To test this hypothesis, we conducted an fMRI experiment involving visuomotor learning and relearning (24 h later), examining changes in both activation and functional connectivity of brain regions that process errors (identified through a parametric modulation approach). Additionally, we investigated changes in intrinsic functional connectivity following the learning and relearning sessions to gain insight into brain changes that may persist beyond the task.

## Methods

### Population

Twenty-four healthy right-handed young adults (age: 29.4 ± 3.6 years old; 11 women) participated in this study. They were free of any neurological or musculoskeletal injuries, and presented normal or corrected-to-normal vision. The study was conducted with the approval of the ethics committee “Comité de Protection des Personnes Nord Ouest IV” under the approval ID-RCB n° 2020-A00268-31. Written informed consent was obtained from all participants.

An a priori power analysis was conducted using G*Power 3.1.9.7 for sample size estimation (Faul et al. 2007), based on data from Standage et al. (2023). This study investigated visuomotor adaptation on two testing days in a sample of 32 subjects, assessing whether the rate of learning increased over the two days (savings). The effect size was d = 1.08 (obtained from t-value using the formula $\:d=\frac{t}{\sqrt{n}};$ Rosenthal 1991), which can be interpreted as a large effect size (Cohen 1988). With a significance criterion of α = 0.05 and power = 0.80, the minimum sample size needed with this effect size was *n* = 7 for a paired t-test. Furthermore, Standage et al. (2023) identified distinct profiles of learners across days, including one profile with poor savings. Using data of this subsample (*n*=10) and using the same parameterization as above, the sample size needed was *n*=24. Thus, our sample size of *n* = 24 should be adequate to reveal savings, even in the least favorable scenario.

### Experimental setup

**Fig. 1:**
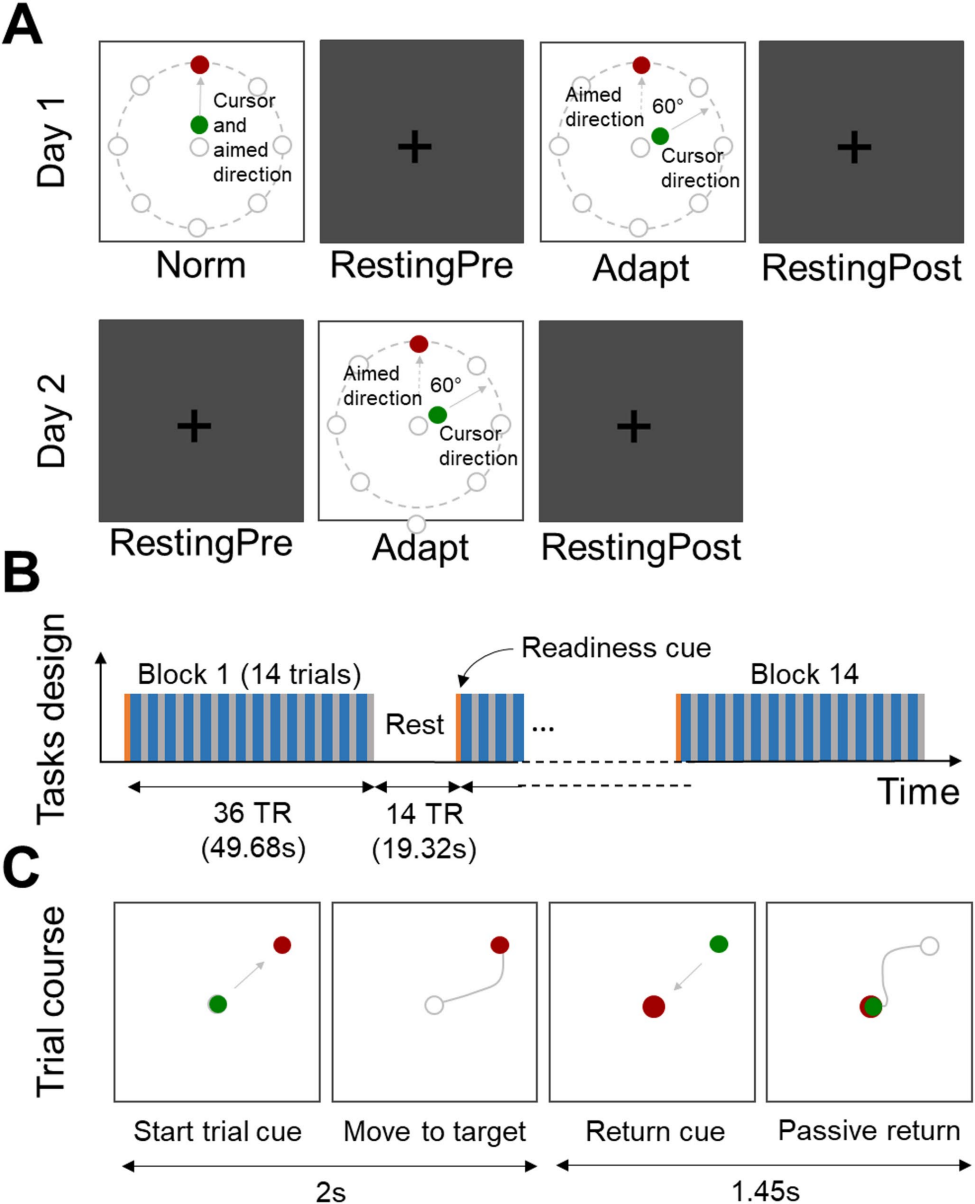
Design of the experiment. (A) Sequence of experimental tasks during the two consecutive days of the experiment. Day1 started with the Norm condition, in which the cursor direction was consistent with participants’ aimed direction. It was followed by a pre-adaptation resting-state, then the adaptation condition, in which the cursor direction was 60° clockwise rotated with respect to the aimed direction. Finally, another resting-state was performed post-adaptation. In Day2, the same sequence was used except the norm condition of Day1. (B) Block design and timing of behavioral tasks (Norm, Adapt-day1 and Adapt-day2) in which 14 blocks of 14 trials were performed. Each block lasted 36 TR (MRI repetition time = 1.38 s), and were intertwined by a resting period of 14 TR. (C) Cues that were seen by participants for each trial, which was divided in two phases, the pointing phase during which the target was displayed in red and the cursor in green, and the passive return phase during which the center of the screen was colored in red. Grey lines are drawn only for comprehension purposes and were not visible by participants

The experiment took place on two consecutive days, which both included an anatomical scan, resting-state runs, and task-based runs (Fig. 1). To reduce head motion and scanner noise, foam padding and earplugs were provided to the participants. During anatomical scans, participants were instructed to lie quietly looking at a black cross, which was displayed on the screen with a grey background to avoid visual fatigue. For the task-based runs, participants held with their right hand a custom-made fMRI-compatible joystick. A Plexiglas adjustable table fixed above their pelvis enabled them to comfortably rest their arm and control the joystick while minimizing movements of the elbow. Participants were instructed to perform the task with their forearm trying to avoid as much as possible large arm movements. Visual stimuli were back-projected onto a monitor (60-Hz frame rate, 3840 × 2160 pixels screen resolution, 40” diagonal, NordicNeuroLab^®^) and viewed through a mirror mounted on the head coil. The task consisted in performing target-directed pointing movements with the joystick that controlled a green cursor (Struber et al., 2021). Each movement started from the center of the screen to one of eight possible targets equally spaced around a virtual circle (radius 20 cm). Each trial started with one of the eight targets becoming red. Participants had to reach the target with the green cursor as fast and as accurately as possible. Once the target was reached, it turned to blue and participants had to maintain the cursor inside the target until the target disappeared and a red circle appeared in the middle of the screen indicating them to passively let the joystick return to its initial position (Fig. 1). Allotted time was 2 s for target reaching and 1.45 s for passive return movement. These specifications allowed us to collect fMRI data during 36 repetition times (TR) per block. The task was implemented using a custom C + + software based on Qt and Measurement Computing© libraries. This software was synchronized with the MRI scanner.

### Experimental conditions

Participants had to adapt to a constant perturbation on two consecutive days (Adapt-day1 and Adapt-day2). A 60° clockwise (CW) rotation angle was maintained between the cursor on the screen and the actual movement performed by the participants. We deliberately chose to not include null trials (i.e., washout) between the two days of adaptation to avoid unwanted anterograde interference and magnify, as much as possible, the extent of savings (Villalta et al. 2015). On day1, a normal, unrotated, movement condition was included before the adaptation condition so as to identify seed regions afterwards used for connectivity analysis (cf. fMRI Processing). Resting-state scans were also acquired on both days immediately before and after the adaptation task (RestingPre-day1, RestingPost-day1, RestingPre-day2 and RestingPost-day2). This was included so as to investigate possible changes in intrinsic connectivity following adaptation. Each task condition included 14 blocks of 49.68 s (36 TR) which started with 1 TR of readiness indicating the beginning of the block, followed by 14 trials of target-directed pointing movement with the cursor for a total of 196 trials per condition. Each block was followed by 19.32 s (14 TR) of rest (Fig. 1).

### Behavioral analysis and modelling

Cursor positions acquired at 2048 Hz were filtered through a dual 4th order low pass Butterworth filter with a 5 Hz cutoff frequency and were then down-sampled at 100 Hz. Beginning of pointing movement was defined as the time the cursor velocity was above 20% of peak velocity. End of pointing movements was defined as the time the cursor entered into the target. Trials in which participants did not reach the target were excluded from further analyses (1.5%, 6.1% and 4.9% of the trials in Norm, Adapt-day1 and Adapt-day2 conditions, respectively). For each trial, the motor performance was quantified by the normalized cumulative error performed by the participant during target-directed pointing. To do so, at each time step, the error was defined as the distance between the actual cursor position and the shortest trajectory (i.e., the straight-line connecting the starting position and the target). Then, all errors were summed (square root of the sum of squared distances) and normalized by reaching duration. This variable was used afterwards in parametric modulation of brain activation (see below).

For each participant, learning was modeled by fitting a state-space model (SSM) to trial-by-trial error data (Diedrichsen et al. 2005; Donchin et al. 2003). Specifically, learning was modeled separately for each day (i.e., for each Adapt condition) using both one-state and two-state SSMs (Albert and Shadmehr 2018). The one-state SSM provided a parsimonious account of adaptation changes across days, while the two-state SSM provided a more detailed characterization by dissociating the contributions of fast and slow learning processes.

Our SSM possessed the same canonical form widely used in previous studies (Albert and Shadmehr 2018; Smith et al. 2006):

1 $$\:\left\{\begin{array}{c}{x}_{n+1}={D}_{n}.A{x}_{n}+B\left(u-{y}_{n}\right)\\\:{y}_{n}={x}_{n}\end{array}\right.\:$$

This formulation applies to both the one-state and two-state versions of the model. In the two-state case, the state vector *x*_*n*_ includes both fast and slow learning components, and the matrices are defined as:

$$\:\:\:\:A=\left[\begin{array}{cc}{a}_{slow}&\:0\\\:0&\:{a}_{fast}\end{array}\right]\:,\:\text{a}\text{n}\text{d}\:B=\left[\begin{array}{c}{b}_{slow}\\\:{b}_{fast}\end{array}\right]$$

In the one-state model, *x*_*n*_​, *A*, and *B* are scalars that represent a single adaptive process.

The model describes how the internal state *x*, which gives rise to the movement output *y*, is updated from trial *n* to trial *n* + 1. This update is driven by the experienced error (*u − y*_*n*_​), where *u* is the magnitude of the imposed perturbation. The matrix (or scalar) *B* determines error sensitivity, i.e., the proportion of the error that contributes to updating the state, thereby controlling the rate of learning from error. The matrix (or scalar) *A* represents retention, encoding how much of the internal state is preserved between trials, or equivalently the extent to which the state decays over time in the absence of error. The model also accounted for additional forgetting during breaks between trial blocks through the decay factor *D*_*n*_ defined as:

$$\:{D}_{n}=\left\{\begin{array}{c}\left[\begin{array}{cc}d&\:0\\\:0&\:d\end{array}\right]\:\text{,}\:\text{i}\text{f}\:\text{t}\text{r}\text{i}\text{a}\text{l}\:\text{n}\:\text{i}\text{s}\:\text{f}\text{o}\text{l}\text{l}\text{o}\text{w}\text{e}\text{d}\:\text{b}\text{y}\:\text{a}\:\text{s}\text{e}\text{t}\:\text{b}\text{r}\text{e}\text{a}\text{k}\:\\\:\left[\begin{array}{cc}1&\:0\\\:0&\:1\end{array}\right],\:\text{otherwise}\end{array}\:,\:\right.$$ (two-state model).

Or simply.

$$\it \it \it \it \:{D}_{n}=\left\{\begin{array}{c}d\:\text{,}\:\text{i}\text{f}\:\text{t}\text{r}\text{i}\text{a}\text{l}\:\text{n}\:\text{i}\text{s}\:\text{f}\text{o}\text{l}\text{l}\text{o}\text{w}\text{e}\text{d}\:\text{b}\text{y}\:\text{a}\:\text{s}\text{e}\text{t}\:\text{b}\text{r}\text{e}\text{a}\text{k}\:\\\:1,\:\text{otherwise}\end{array}\:,\:\right.$$ (one-state model).

Parameters $\:A$, $\:B$, $\:{x}_{0}$ and $\:D$ were estimated by optimization. To do so, the mean square error between actual error and prediction error $\:u-{y}_{n}$ was minimized (using Matlab fmincon function and interior-point algorithm with optimality and constraint tolerances set to 10^− 15^) to obtain the optimal parameter values (LMSE algorithm). Our constrained search parameter space was defined by the following lower and upper bounds: $\:A\in\:\left[0;1\right]$, $\:B\in\:\left[0;1\right]$, $\:{x}_{0}\in\:\left[-2u;2u\right]$ and $\:D\in\:\left[0.1;30\right]$. In the case of the two-state model, additional linear inequality constraints were specified:

$$\:\begin{array}{c}{A}_{s}\ge\:{A}_{f}+0.001\:\\\:{B}_{f}\ge\:{B}_{s}+0.001\end{array}$$

These constraints ensure that the slow state was retained more strongly from trial to trial than the fast state, and that the fast state learned more rapidly from error than the slow state (Albert and Shadmehr 2018).

We also derived an extra parameter from the model equation, the asymptotic motor error, defined as (Albert et al. 2021):

2 $$ \:y_{{\infty \:}} = \:\frac{B}{{1 - A + B}}\:.\:u\,\,\,\left( {{\text{one - state model}}} \right) $$

Or.

3 $$ \:y_{{\infty \:}} = \left[ {\begin{array}{*{20}c} 1 & 1 \\ \end{array} } \right].\:\:\left[ {\begin{array}{*{20}c} {1 - A_{f} + B_{f} } & {\:B_{f} } \\ {\:B_{s} } & {\:1 - A_{s} + B_{s} } \\ \end{array} } \right]^{{ - 1}} \:.\:\:\left[ {\begin{array}{*{20}c} {B_{f} } \\ {\:B_{s} } \\ \end{array} } \right].u\,\,{\text{(two - state model)}} $$

This parameter reflects the steady-state error level after prolonged exposure to the perturbation, offering insights into the ultimate effectiveness of adaptation.

Differences in model parameters between days 1 and 2 were assessed using paired *t*-tests with a level of significance set at *p* < 0.05. The amount of savings was quantified as the difference in B values between days 1 and 2, any significant increase of B reflecting savings. Effect sizes (Cohen’s d for paired samples) were also calculated. Analysis and statistical testing were performed using Matlab (R2018b).

### MRI acquisition

MRI data were acquired at 3T (Achieva dStream 3.0T TX, Philips, NL) with a 32-channel head coil at IRMaGe MRI facility (Grenoble, France). To account for different head placements into the MRI, structural T1-weighted images were acquired at the beginning of each day, using a Magnetization-Prepared Rapid Acquisition Gradient Echoes (MPRAGE) (TI = 900ms, TR/TE = 8.2 ms/4.7 ms, 220 slices, in-plane resolution = 1 × 1 mm, slice thickness = 1 mm, flip angle = 8°, field of view = 256 × 256 × 220 mm^3^). Compressed SENSE with acceleration factor 4.4 was used. Resting scans functional blood-oxygen level-dependent (BOLD) images were collected using a T2*- echo-planar sequence with multiband acceleration of 3 and SENSE factor of 2 (TR/TE = 1620/30 ms, voxel size = 2.25 × 2.25 × 2 mm^3^, gap = 0.25 mm, 69 slices, field of view = 216 × 216 × 155 mm^3^, flip angle = 70°, 350 volumes). Task-based functional BOLD images were obtained with a T2*- echo-planar sequence with multiband acceleration of 3 and SENSE factor of 2 (TR/TE = 1380/30 ms, voxel size = 2.5 × 2.5 × 2.25 mm^3^, gap = 0.25 mm, 63 slices, field of view = 200 × 218 × 157 mm^3^, flip angle = 70°, 700 volumes). To correct for magnetic field inhomogeneity during data preprocessing, we also acquired a pair of spin-echo images before each BOLD scan (same specifications as above for task-based or resting), with reversed phase encoding direction.

### MRI preprocessing

After conversion into a BIDS dataset (Gorgolewski et al. 2016), preprocessing was performed using FMRIPREP version 20.2.6 (Esteban et al. 2019; RRID: SCR_016216), a Nipype (Gorgolewski et al. 2017; RRID: SCR_002502) based tool. Each T1w (T1-weighted) volume was corrected for INU (intensity non-uniformity) using N4BiasFieldCorrection v2.1.0 (Tustison et al. 2010) and skull-stripped using antsBrainExtraction.sh v2.1.0 (using the OASIS template). Brain surfaces were reconstructed using recon-all from FreeSurfer v6.0.1 (Dale et al. 1999; RRID: SCR_001847), and the brain mask estimated previously was refined with a custom variation of the method to reconcile ANTs-derived and FreeSurfer-derived segmentations of the cortical gray-matter of Mindboggle (Klein et al. 2017; RRID: SCR_002438). Spatial normalization to the ICBM 152 Nonlinear Asymmetrical template version 2009c (Fonov et al. 2009; RRID: SCR_008796) was performed through nonlinear registration with the antsRegistration tool of ANTs v2.1.0 (Avants et al. 2008; RRID: SCR_004757), using brain-extracted versions of both T1w volume and template. Brain tissue segmentation of cerebrospinal fluid (CSF), white-matter (WM) and gray-matter (GM) was performed on the brain-extracted T1w using fast (Zhang et al. 2001; FSL v5.0.9, RRID: SCR_002823).

Functional data was slice time corrected using 3dTshift from AFNI v16.2.07 (Cox 1996; RRID: SCR_005927) and motion corrected using mcflirt (FSL v5.0.9, Jenkinson et al. 2002). Distortion correction was performed using an implementation of the TOPUP technique (Andersson et al. 2003) using 3dQwarp (AFNI v16.2.07). This was followed by co-registration to the corresponding T1w using boundary-based registration (Greve and Fischl 2009) with nine degrees of freedom, using bbregister (FreeSurfer v6.0.1). Motion correcting transformations, field distortion correcting warp, BOLD-to-T1w transformation and T1w-to-template (MNI) warp were concatenated and applied in a single step using antsApplyTransforms (ANTs v2.1.0) using Lanczos interpolation.

ICA-based Automatic Removal of Motion Artifacts (ICA-AROMA; Pruim et al. 2015) was performed on the preprocessed functional data after spatial smoothing with an isotropic Gaussian kernel of 6 mm FWHM (full-width half-maximum). Noise components were partially regressed in the internal regression step of ICA-AROMA, which constitutes an effective strategy to abolish motion-related variance for relatively low cost in terms of data loss (Ciric et al. 2017; Parkes et al. 2018). Additionally, region-wise global signals within WM and CSF were computed.

Many internal operations of FMRIPREP use Nilearn (Abraham et al. 2014; RRID: SCR_001362), principally within the BOLD-processing workflow. For more details of the pipeline see https://fmriprep.readthedocs.io/en/20.2.6/workflows.html.

### fMRI processing

Task-based and resting-state (participant-level and group-level) analyses were performed using custom-made (batch syntax) Matlab routines (R2018b) running SPM12 toolbox (https://www.fil.ion.ucl.ac.uk/spm/) and CONN toolbox (https://web.conn-toolbox.org/), respectively.

#### GLM analysis

Brain activation and functional connectivity during the adaptation sessions were assessed using standard and beta series (least squares-all method) GLMs, respectively (Rissman et al. 2004). Beta series GLM differ from standard GLM in that the model is fitted to the fMRI data trial-wise, and not condition-wise. This creates time series of beta estimates which can be subsequently correlated to estimate functional connectivity. Here, the GLM design matrix included movement trials, each trial being represented as a boxcar function time-locked to the onset of movement and convolved with SPM canonical hemodynamic response function (HRF). Trials were represented all together in a single column of the design matrix in standard GLM while they were represented separately in as many columns as there were trials in beta series GLM. A parametric modulator was also entered into the design matrix to model the linear effect relating the evoked-response during each trial to the magnitude of movement error. This modulator was entered the same way as the movement trials in the GLM, i.e., in a single column for standard GLM and in as many columns as there were trials in beta series GLM. This parametric modulator was demeaned to avoid collinearity issues prior to convolution with the HRF (Mumford et al. 2015). The WM and CSF time series were further entered into the design matrix to serve as nuisance variables.

As for standard GLM, individual beta maps of the parametric modulator were then entered into a second-level analysis. One-sample t-tests revealed brain regions whose activation related to movement errors during the adaptation sessions. A two-sample t-test assessed whether activation of these regions related to errors was different between adaptation and re-adaptation sessions.

As for beta series GLM, we extracted the mean beta series of the parametric modulator from four regions of interest (ROIs), including the primary motor cortex (M1), the primary somatosensory cortex (S1), and cerebellar sensorimotor regions, specifically lobules VI and VIIIb of the cerebellum. As mentioned in introduction, these regions appear to be key regions for the storage of motor memories. ROIs were 6 mm radius spheres built after running a standard GLM on the Norm condition and extracting coordinates of local maxima of the group-level (one-sample t-test) activation map (Norm versus implicit baseline). ROIs were centered at MNI coordinates [-38, -18, 62] for left M1, [-34, -30, 52] for left S1, [28, –48, -26] for right cerebellar lobule VI, and [18, –60, -54] for right cerebellar lobule VIIIb (Figure S1). The fact that ROIs were obtained from a condition (i.e., Norm) other than the condition under study (i.e., Adapt) was done to contain the risk of circular analysis. As a second step, the beta series of the parametric modulator extracted from each ROI was correlated (Pearson) with the beta series of the parametric modulator from every voxel in the rest of the brain. We used the doBetaSeries function from Andy’s brain blog (http://andysbrainblog.blogspot.com/2014/06/beta-series-analysis-in-spm.html). This yielded correlation maps that were finally converted to z-score maps and entered into the second-level analysis. One-sample t-tests revealed co-activation, i.e. functional connectivity, patterns of the ROIs during the adaptation sessions. It is worth noting, given the methodology described above, that these patterns reflect similarities between the ROIs and other brain regions in their responses to error during the task, referred to throughout the manuscript as error-related or error-modulated connectivity. Finally, two-sample t-tests assessed whether the error-modulated co-activation patterns of the ROIs were different between adaptation and re-adaptation sessions.

#### Resting-state connectivity

Functional connectivity before and after adaptation sessions was scrutinized using seed-to-voxels and independent component (ICA) approaches. Seed-to-voxels maps were computed as the Fisher-transformed bivariate correlation coefficients between denoised average BOLD time series computed across all the voxels within each ROI presented above and each individual voxel of denoised BOLD time series in the brain. Group-level ICA followed Calhoun’s general methodology (Calhoun et al. 2001) and estimated 40 temporally coherent networks from the resting-state fMRI data combined across all subjects and conditions. The BOLD signal from every timepoint and voxel in the brain was concatenated across subjects and conditions along the temporal dimension. A singular value decomposition of the z-score normalized BOLD signal (subject-level SVD) with 64 components separately for each subject and condition was used as a subject-specific dimensionality reduction step. The dimensionality of the concatenated data was further reduced using a singular value decomposition (group-level SVD) with 40 components, and a fast-ICA fixed-point algorithm with hyperbolic tangent (G1) contrast function was used to identify spatially independent group-level networks from the resulting components. GICA3 back-projection was then used to compute ICA maps associated with these same networks separately for each individual subject and condition. Finally, we identified the network we were interested in, namely the sensorimotor network, using the correlational spatial match-to-template tool of CONN. Any difference that may have occurred between the resting state conditions were examined from F-tests run on seed-to-voxel and ICA (sensorimotor) maps.

#### Thresholding of statistical maps

Maps were thresholded using a cluster-forming threshold *p* < 0.001 and a cluster-extent based threshold *p* < 0.05 family-wise error rate (FWER) corrected, according to established recommendations (Eklund et al. 2016; Woo et al. 2014). Cluster-extent threshold was estimated using the Gaussian random field method (Worsley et al. 1996), as implemented in SPM 12 and CONN 19.

## Results

### Behavior

**Fig. 2:**
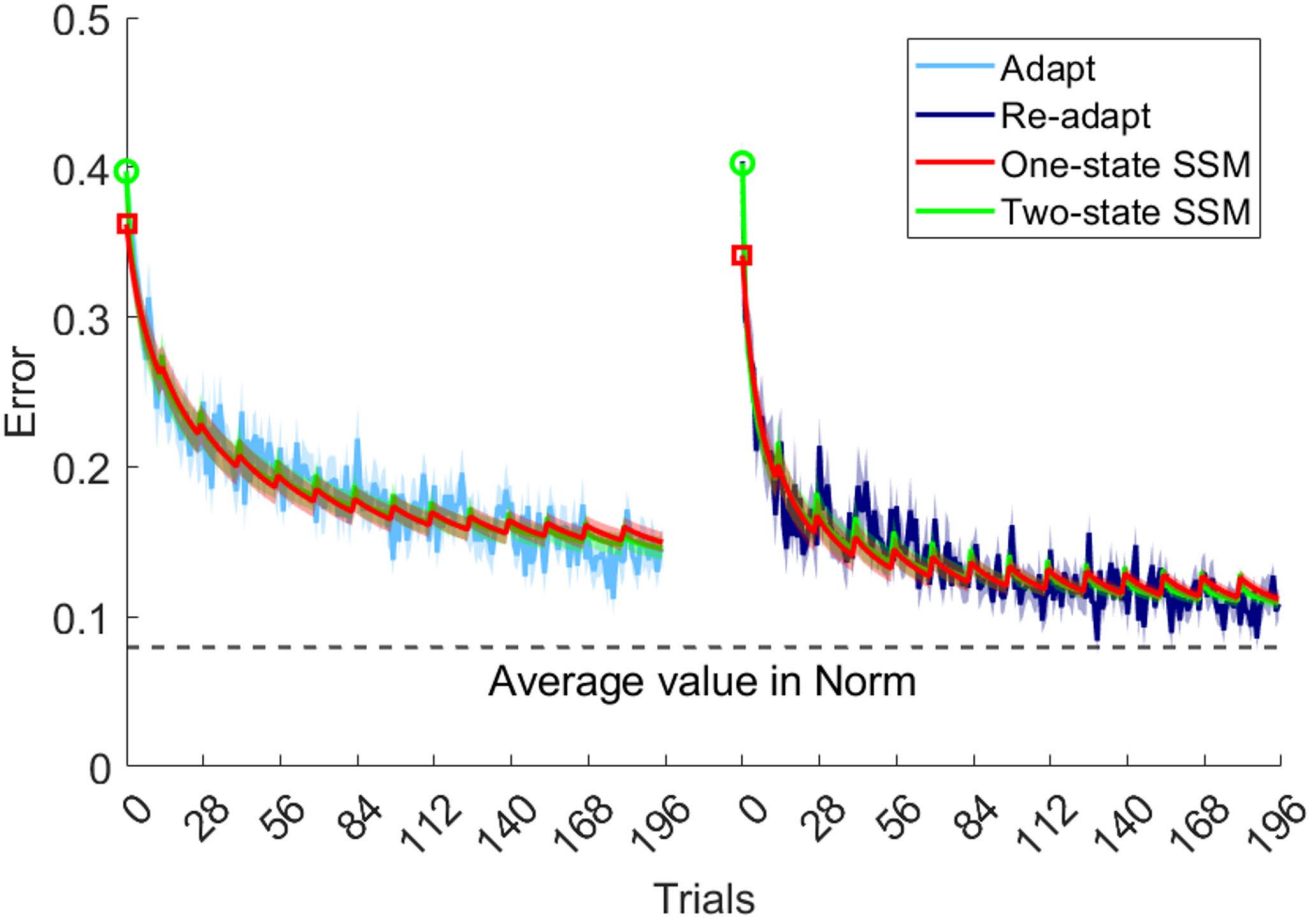
Behavioral data and model simulations. Evolution of movement error across trials during Adapt-day1 (light blue) and Adapt-day2 (dark blue) conditions, and as predicted by the state space models (SSM). Curves represent mean ± standard error across subjects

As shown in Fig. 2, error during adaptation sessions followed a stereotypical pattern, with a rapid decrease during the initial trials, followed by a more gradual decrease thereafter. As expected, simulation using one-state and two-state models reproduced this error pattern well.

Paired t-tests on the estimated SSM parameters identified changes between day1 and day2 (Table 1). The one-state SSM parameters showed that error sensitivity, which controls how quickly the state updates based on previous errors, significantly increased from day1 to day2 (t_23_=− 2.2232, *p* = 0.0363, d=-0.46), while the retention factor, which reflects how the state decays over time, remained consistent across days. Hence, there was a greater amount of learning from error during relearning, consistent with the concept of savings, while the rate of forgetting between trials remained unchanged. Importantly, there was no difference in initial error between day1 and day2 (as represented by markers at trial 0 in Fig. 2), indicating that the observed savings was not influenced by memory retrieval processes that could have biased its estimation.

The findings from the two-state SSM analysis were consistent with those of the one-state analysis, revealing similar retention rates across days 1 and 2, along with a selective increase in the learning rate that was confined to the slow process (t_23_=− 2.2879, *p* = 0.0317, d=-− .47). This suggests that savings were driven by the slow learning component.

Finally, asymptotic performance improved from Day 1 to Day 2, evidenced by a significant reduction in asymptotic error in the one-state SSM (t_23_ = 2.83, *p* = 0.0095, d = 0.58), and a marginally significant reduction in the two-state SSM (t_23_ = 2.01, *p* = 0.0563, d = 0.41). This improved performance was driven by an increased learning rate between days, while the forgetting rate remained unchanged (cf. Equation 2a & 2b).

**Table 1:** State space model (SSM) parameter values

			Retention factor [A]	Error sensitivity [B]	Asymptote [y_∞_]	AICc
One-state SSM	Day 1		0.995 ± 0.006	0.052 ± 0.064	0.123 ± 0.052	-503.04 ± 71.58
	Day 2		0.992 ± 0.008	0.106 ± 0.101^(0.0363)^	0.089 ± 0.036^(0.0095)^	-529.76 ± 85.73
Two-state SSM	Day 1	Slow	0.994 ± 0.008	0.043 ± 0.051	0.109 ± 0.065	-501.67 ± 85.83
		Fast	0.502 ± 0.310	0.175 ± 0.244		
	Day 2	Slow	0.992 ± 0.008	0.084 ± 0.080^(0.0317)^	0.083 ± 0.036^(0.0563)^	-533.32 ± 74.29
		Fast	0.482 ± 0.301	0.197 ± 0.228		

Asymptotic error values and the quality of model fits, quantified by the Corrected Akaike Information Criterion (AICc), are also shown. Values are mean ± SD. Significant p-values from the Day 1 vs. Day 2 comparison are shown in parentheses.

### Activation of brain regions responsive to errors

**Fig. 3:**
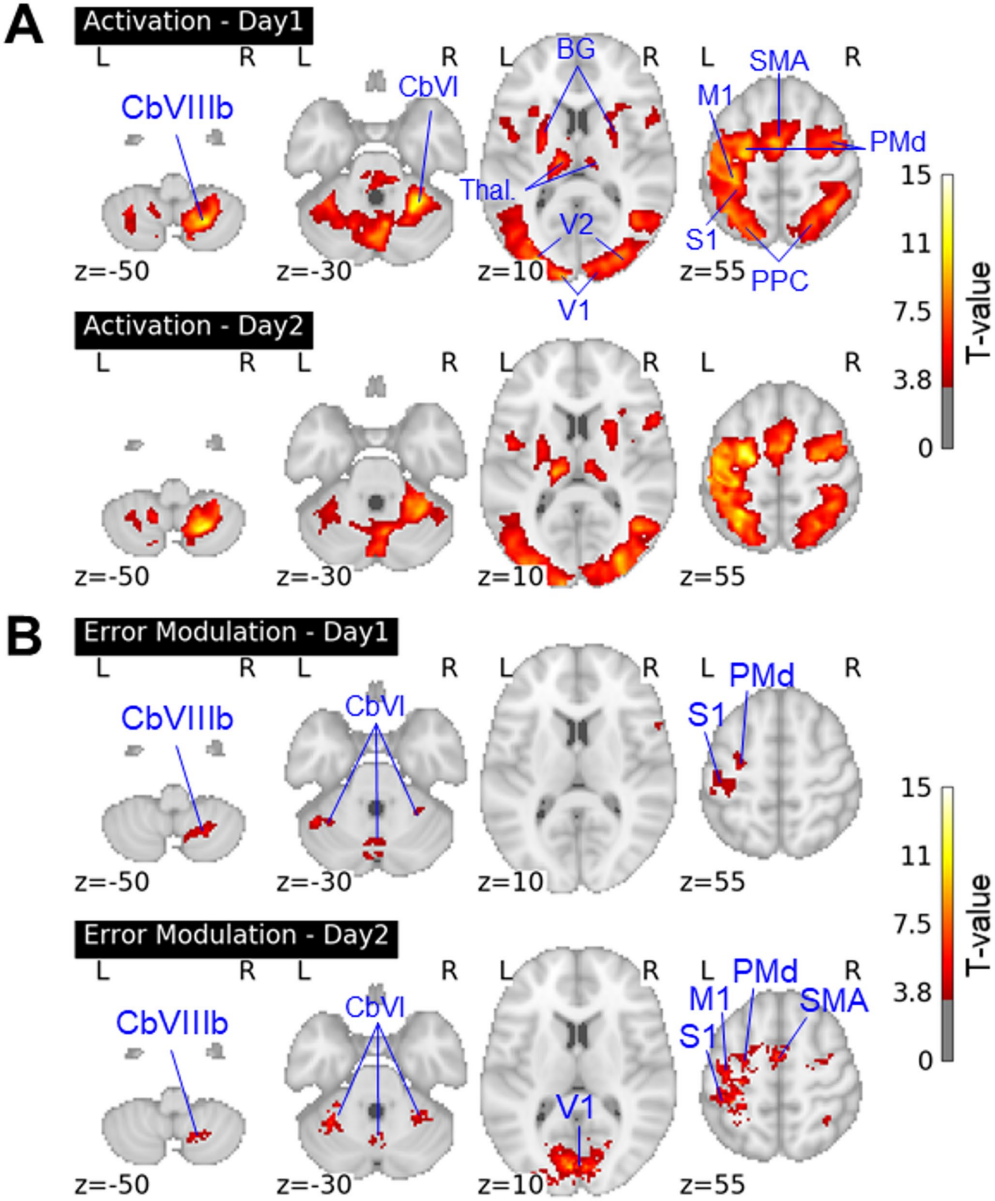
**Brain activation.** (A) Activation maps during adaptation (vs. implicit baseline) on Day 1 and Day 2. (B) Error-related positive modulation of activation

M1: primary motor cortex; S1: primary somatosensory cortex; SMA: supplementary motor area; PMd: dorsal premotor cortex; PPC: posterior parietal cortex; BG: basal ganglia; Thal.: thalamus; V1: primary visual cortex; V2: secondary visual cortex; CbVI: cerebellar lobule VI; CbVIIIb: cerebellar lobule VIIIb. R = right hemisphere; L = left hemisphere. x, y, z: coordinates in MNI space.

Regional patterns of activation obtained during the adaptation sessions are reported in Fig. 3. For the sake of providing an overall picture of what happened at the brain level during adaptation, we first reported brain activation during the adaptation trials regardless of whether activation was modulated by error magnitude (Fig. 3A). Activated regions on day1 and day2 included, as expected for a right-hand movement, the sensorimotor parcels (lobules V, VI, and VIIIb) of the right cerebellum, the left sensorimotor cortical areas (primary motor cortex – BA4, primary somatosensory cortex – BA1, dorsal premotor cortex – BA6, supplementary motor area – BA6), and regions of the left posterior parietal cortex (superior parietal lobule – BA5 and BA7, and extending a bit into the supramarginal gyrus – BA40). Activation foci were also found in the right dorsal premotor cortex, the right superior parietal lobule, and the left sensorimotor cerebellum (lobules V, VI, and VIIIb). Given the visuomotor nature of the task, activation was also observed in the right and left primary and secondary visual (BA17 and BA18, respectively) cortices. Finally, subcortical regions were also part of the activation network, including the right and left basal ganglia (putamen) and thalamus. There was no activation difference between days 1 and 2.

A subset of regions listed above demonstrated a positive parametric modulation of activation by error magnitude on days 1 and 2 (Fig. 3B). Activation foci were located in the right lobule VIIIb and the right and left lobule VI of the cerebellum, in the primary visual cortex, and in the left primary somatosensory and dorsal premotor cortices. Activation was also related to errors in the primary motor cortex and the supplementary motor area on day2. Comparison between days did not reveal any difference in error modulation.

### Connectivity between brain regions responsive to errors

**Fig. 4:**
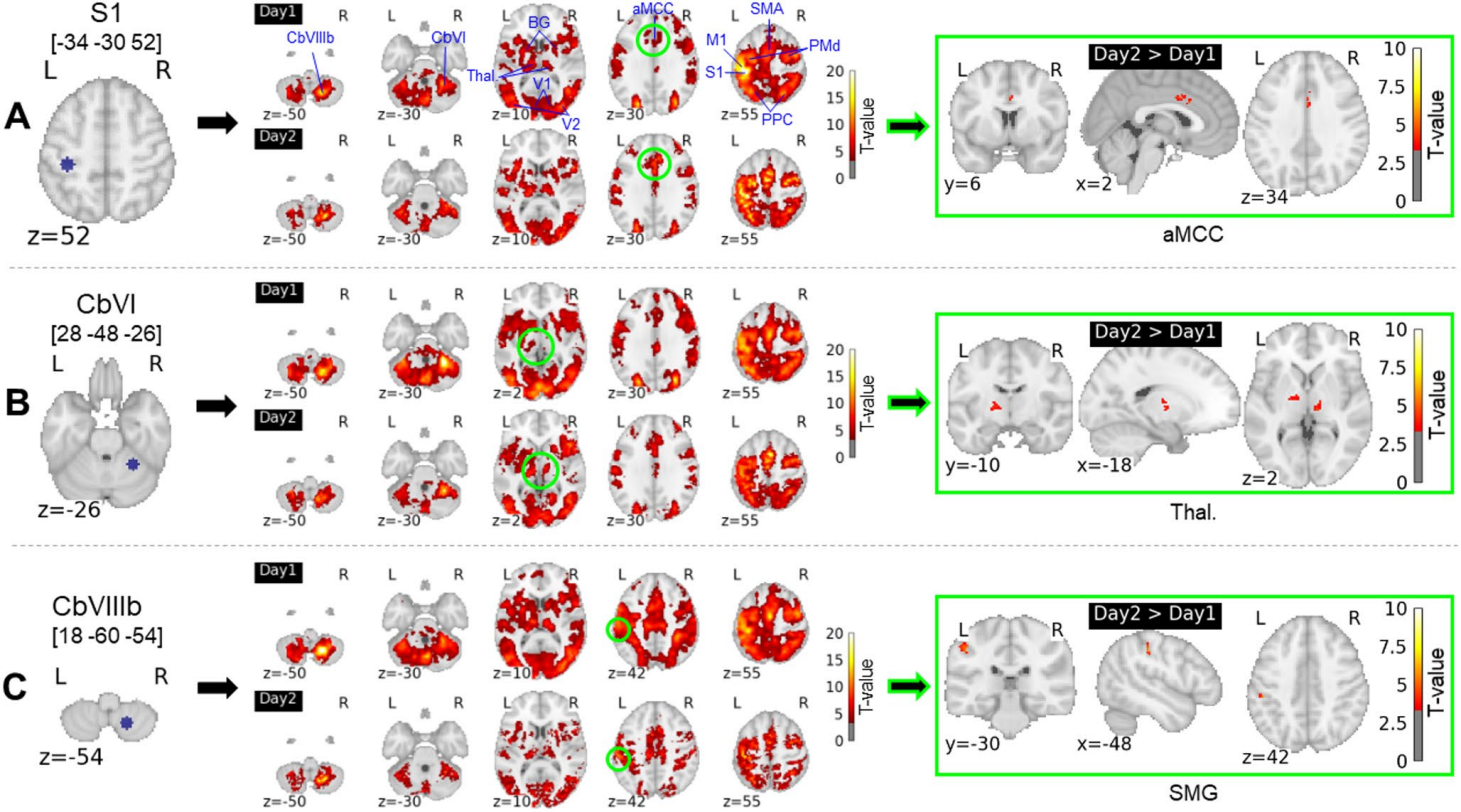
Error-modulated ROI-to-voxel co-activation patterns. The contrast day2 > day1 reveals regions whose connectivity with ROIs was more strongly modulated by error on day2 compared to day1. These regions are situated on the connectivity maps using green open circles

The error-modulated co-activation patterns looked roughly similar for all ROIs and delineated the same error-related network of interconnected regions that spread over the cerebral cortex, the cerebellum, the basal ganglia and the thalamus (Figs. 4 & S2). Specifically, they were composed of sensorimotor (BA1, 4, 6), posterior parietal (BA5, 7, 40) and visual (BA17, 18) brain regions, sensorimotor cerebellar territories (lobules V, VI and VIIIb), as well as basal ganglia and thalamus in both the left and right hemispheres. Interestingly, there were also areas located more anteriorly in the frontal lobe, especially in the anterior midcingulate cortex (BA 24, 32) and in the lateral prefrontal cortex (BA9, 44).

**Fig. 5:**
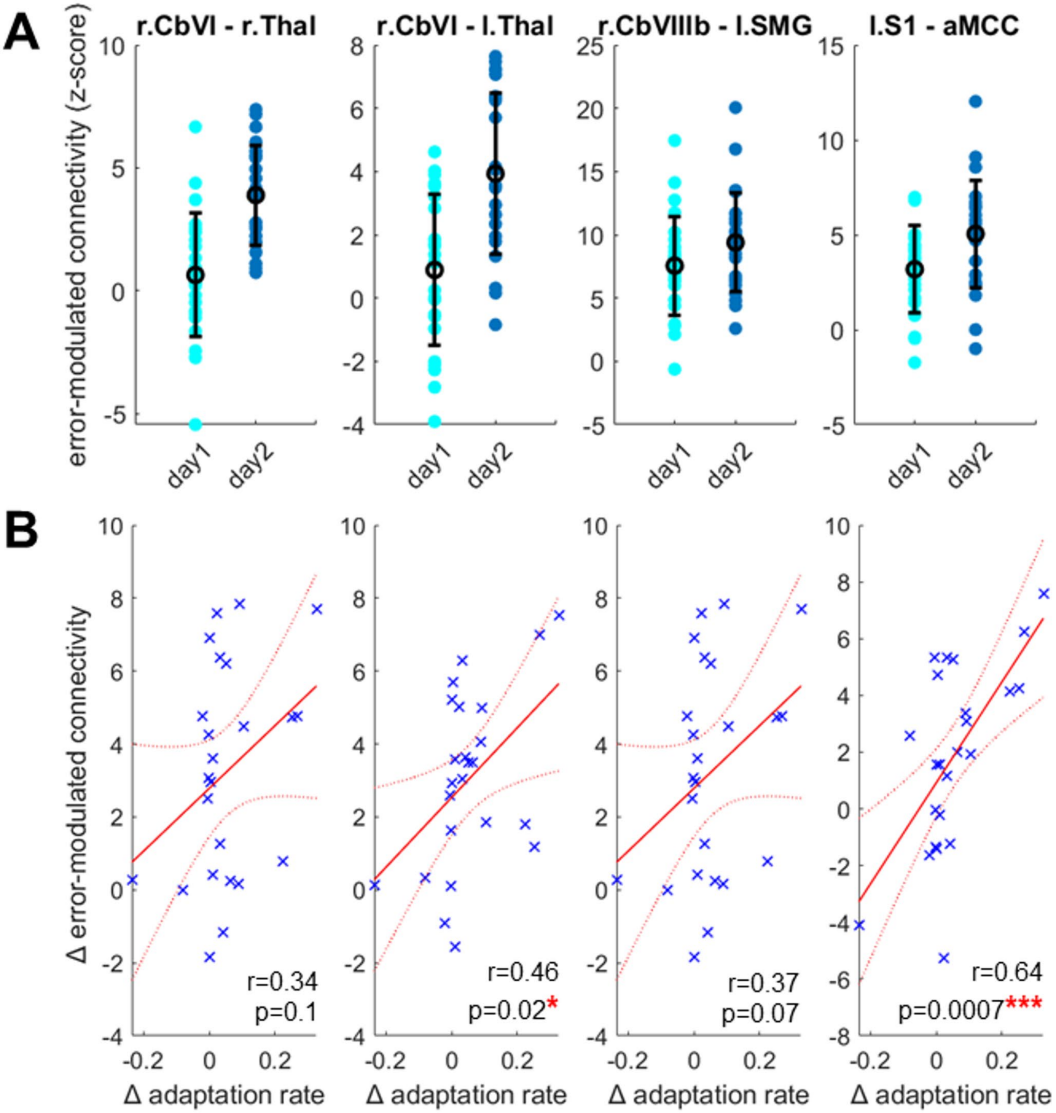
Relationship between error-modulated interregional co-activation and savings. (A) Pathways whose connectivity was more strongly modulated by error in day2 than in day1. (B) Relationship between changes in error-modulated co-activation and savings. Savings is represented as change in adaptation rate

S1: primary somatosensory cortex; aMCC: anterior midcingulate cortex; SMG: supramarginal gyrus; Thal: thalamus; CbVI: cerebellar lobule VI; CbVIIIb: cerebellar lobule VIIIb. r = right hemisphere; l = left hemisphere.

Importantly, the functional connectivity was more strongly modulated by error on day2 compared to day1, between (i) the left primary somatosensory cortex (S1) and the posterior portion of the rostral cingulate zone (RCZp), located in the anterior midcingulate cortex (MNI coord. = [2, 6, 34], n voxels = 39, pFWE-corr = 0.05; Figs. 4A and 5A); (ii) the right cerebellar lobule VI and the left ventrolateral thalamus, which aligns well with the ventral intermediate (Vim) nucleus (MNI coord. = [-18, -10, 2], n voxels = 49, pFWE-corr = 0.04; Figs. 4B and 5A and figure S3); (iii) the right cerebellar lobule VI and the right ventrolateral thalamus, covering the Vim nucleus (MNI coord. = [10, -22, 2], n voxels = 35, marginal effect with pFWE-corr = 0.161 but qFDR-corr: 0.044; Figs. 4B and 5A and figure s3), and (iv) right cerebellar lobule VIIIb and left supramarginal gyrus (MNI coord. = [-48, -30, 42], n voxels = 38, pFWE-corr = 0.031; Figs. 4C and 5A).

aMCC: anterior midcingulate cortex; M1: primary motor cortex; S1: primary somatosensory cortex; SMA: supplementary motor area; PMd: dorsal premotor cortex; PPC: posterior parietal cortex; SMG: supramarginal gyrus; BG: basal ganglia; Thal.: thalamus; V1: primary visual cortex; V2: secondary visual cortex; CbVI: cerebellar lobule VI; CbVIIIb: cerebellar lobule VIIIb. R = right hemisphere; L = left hemisphere. x, y, z: coordinates in MNI space.

Regarding the pathways identified above, between left S1 and RCZp, and between right cerebellar lobule VI and ventrolateral left thalamus, the increase in error-modulated functional connectivity from day1 to day2 positively correlated with the increased one-state learning rate across individuals (*r* = 0.64, *p* = 0.0007 and *r* = 0.46, *p* = 0.02, respectively; Fig. 5B), indicating that enhanced error-related connectivity was associated with savings. Note that these two correlations remained significant when adjusting FDR (4 comparisons) using the Benjamini-Hochberg procedure. On the other hand, regarding the other two pathways between right cerebellar lobule VI and right thalamus and right cerebellar lobule VIIIb and left supramarginal gyrus, there was only a tendency for a positive correlation between increased connectivity and increased learning rate (*r* = 0.34, *p* = 0.1 and *r* = 0.37, *p* = 0.07, respectively; Fig. 5B). Finally, we also examined the relationship between increased error-modulated functional connectivity and savings as measured by the enhanced learning rate of the slow component in the dual-state model, but found no significant correlation.

### Changes in intrinsic functional connectivity

**Fig. 6:**
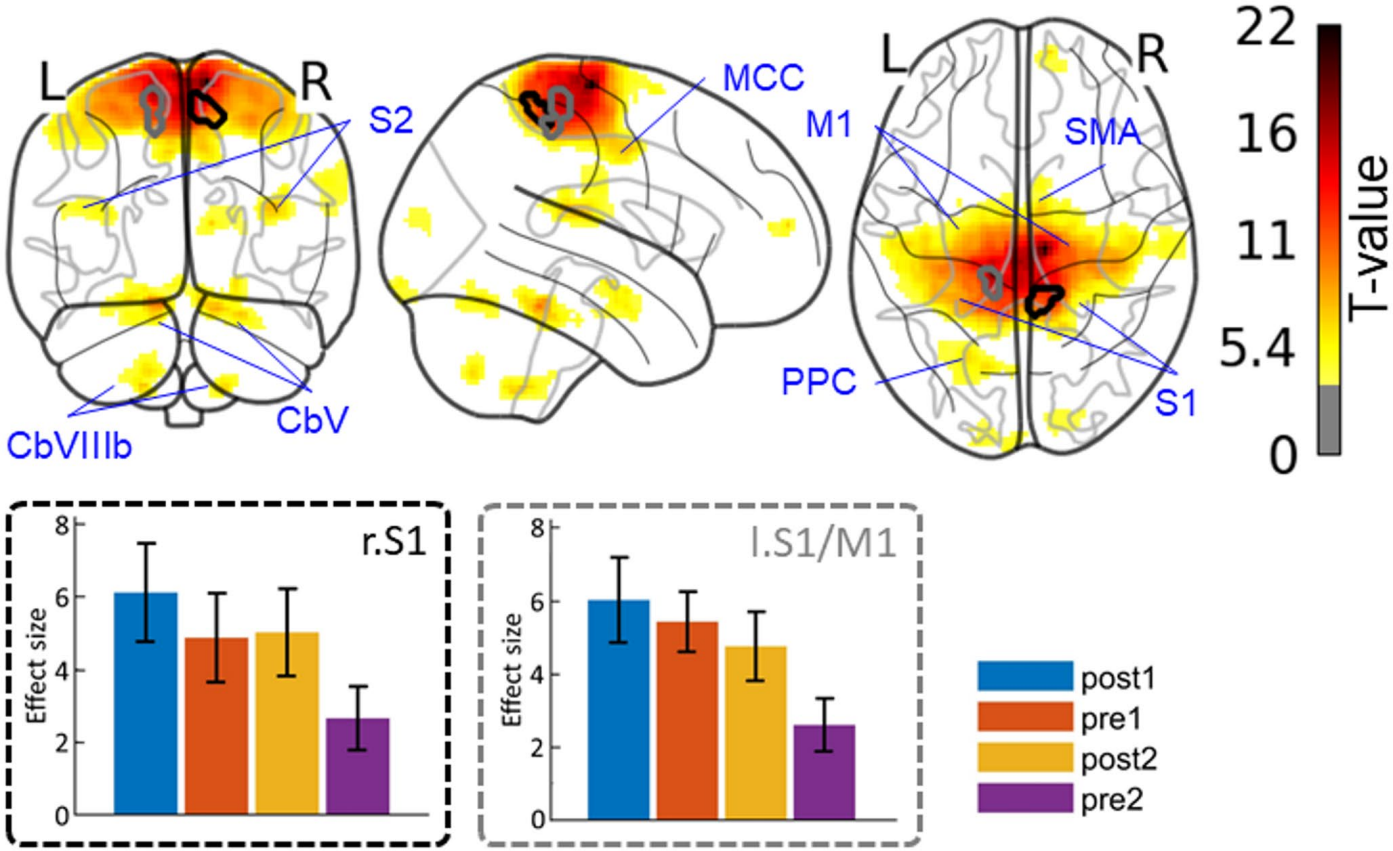
Sensorimotor ICA map during the conditions of resting-state. Clusters that showed increased connectivity following adaptation in both day1 and day2 are represented with black and grey contours

M1: primary motor cortex; S1: primary somatosensory cortex; SMA: supplementary motor area; PPC: posterior parietal cortex; MCC: midcingulate cortex; CbV: cerebellar lobule V; CbVIIIb: cerebellar lobule VIIIb; r.: right hemisphere; l.: left hemisphere.

One-way within-subjects ANOVA run on seed-to-voxels connectivity maps did not reveal any difference of intrinsic connectivity between the resting state conditions (i.e., pre1, post1, pre2, post2). As regards the ICA-based analysis, it was delineated a “sensorimotor” network that roughly corresponded to the network seen in adaptation, which spread over sensorimotor (i.e., supplementary motor area, primary motor cortex, primary and secondary sensorimotor cortices, anterior mid-cingulate cortex, motor territories of the cerebellum) and associative (i.e., posterior parietal cortex) brain regions (Fig. 6). One-way within-subjects ANOVA revealed two clusters of that network that showed connectivity changes between the resting state conditions: one cluster belonged to the right primary somatosensory cortex (MNI coord. = [8, − 44, 60], n voxels = 85, pFWE-corr = 0.027) and the other cluster was located at the boundary between the left primary motor cortex and the left primary somatosensory cortex (MNI coord. = [− 14, − 32, 64], n voxels = 84, pFWE-corr = 0.029). As shown in Fig. 6, contrast estimates from these clusters suggest that the significant effect found in the ANOVA was due to an increased connectivity after adaption on both day 1 (i.e., pre1 vs. post1) and day 2 (i.e., pre2 vs. post2), as well as a decreased connectivity from day 1 to day 2 (i.e., post1 vs. pre2). However, none of these connectivity changes were found to be related to re/learning properties.

## Discussion

The present study demonstrated that individuals with greater savings on a visuomotor learning task exhibited a larger increase in connectivity between regions of a cerebello-thalamo-cortical network that responds to movement errors. This finding suggests that a more rapid re-adaptation to perturbations occurs through a mechanism that enhances communication among a network of distributed brain regions involved in error processing.

Specifically, we found that the amount of savings was associated with increased connectivity in two pathways: one between the primary somatosensory cortex and the posterior portion of the rostral cingulate zone (RCZp) that lies in the anterior midcingulate cortex (aMCC), and another between the sensorimotor cerebellum (lobule VI) and the ventrolateral thalamus. All four regions are known to be involved in a broad prediction-error monitoring system, processing errors that arise from mismatch between expected and actual outcomes, especially in sensorimotor contexts, and integrating them to adapt action. Previous fMRI studies have shown that RCZp/aMCC responds selectively to error feedback during motor tasks (Hester et al. 2008; Mars et al. 2005). This selective role was further confirmed by intracerebral recordings in humans, where RCZ activity was observed only following erroneous actions, not correct ones (Bonini et al. 2014). Based on these findings, the authors proposed a hierarchical organization within the medial frontal cortex, in which the supplementary motor area monitors action execution, while the RCZ processes performance errors requiring behavioral correction. Supporting this, an EEG study of reaching movements reported a frontocentral event-related potential modulated by unexpected kinematic errors, resembling the feedback-related negativity typically linked to reward prediction errors (Torrecillos et al. 2014). These findings suggest that medial frontal areas, including the RCZ, share neural mechanisms for processing both sensory and reward-based prediction errors. Further evidence from single-cell recordings in primates shows that neuronal activity in the RCZ/aMCC is modulated by reward expectations, with heightened responses when outcomes are worse than expected (Amiez et al. 2005). In summary, the RCZ plays a central role in monitoring errors related to motor execution, sensory discrepancies, and reward outcomes (Ullsperger et al. 2014). Beyond the RCZ, the other brain regions, namely the primary somatosensory cortex, ventrolateral thalamus, and cerebellum, are also key components of this error-monitoring network. Thalamic activity, particularly in the ventrolateral nucleus which is interconnected with the cerebellum and motor cortex, increases during motor adaptation and reflects changes in hand perception, supporting its role in learning from sensory prediction errors (Mahdavi et al. 2024). The motor cerebellum is well-established as a critical structure for computing internal models and representing sensory prediction errors that arise during movement (Brooks et al. 2015; Hull 2020; Izawa et al. 2012; Schlerf et al. 2012; Tseng et al. 2007). Additionally, the primary somatosensory cortex has been shown to receive predictive information about motor output prior to sensory feedback (Umeda et al. 2019), and its inhibition disrupts motor adaptation (Mathis et al. 2017), supporting its role in encoding sensory prediction errors and updating internal models accordingly. Taken together, our findings further support the view that these regions are part of an interconnected network that encodes movement error and adaptively guides behavior. Of course, movement error was genuinely composite in our visuomotor adaptation paradigm, likely including both sensory and reward prediction errors. Indeed, altered visual feedback during reaching movements disrupts the internal model’s predictions about the sensory consequences of motor commands (i.e., sensory prediction error), but also violates expectations about the reward associated with hitting the target (i.e., reward prediction error). This overlap has been highlighted in prior visuomotor adaptation studies, which show that a single perturbation can give rise to a cascade of interrelated error signals (Izawa and Shadmehr 2011; Morehead and Orban De Xivry, 2021). We therefore cannot be specific about the precise nature of the errors that were represented in the connectivity between the four regions.

The current finding that savings relates to cerebellar-thalamic connectivity complements a previous study on locomotor adaptation, which found that baseline cerebellar-thalamic connectivity at rest predicted the amount of savings (Mawase et al. 2017). That study also reported that thalamocortical connectivity was associated with recall, defined as a bias in initial error during relearning, a pattern not observed in our data, where savings was unbiased. Thus, our findings replicate their cerebellar-thalamic result using a task-based functional connectivity approach. Additional evidence supports the thalamus’s role in learning from error: patients with essential tremor who underwent thalamic VIM stimulation or thalamotomy, both of which disrupt thalamic processing, exhibited impaired motor adaptation (Chen et al. 2006). Similarly, increased activity in cerebellar lobule VI and the thalamus has been shown to correlate with the degree of savings during motor task repetition (Debas et al. 2010). Together, these studies and ours suggest that the speed at which individuals learn from errors depends in part on the functional activity and connectivity of the cerebellum and thalamus, with stronger cerebellar-thalamic coupling facilitating more rapid adaptation.

The cerebellum is nevertheless connected with contralateral cortical sensorimotor regions through the ventrolateral thalamus, with a notable role played by the Vim nucleus (Akram et al. 2018; Darian-Smith et al. 1990; Dum and Strick 2003). Although we did not observe thalamocortical projections, this absence may simply reflect insufficient statistical power. Indeed, a sample size of 24 can be considered slightly underpowered (detecting a moderate bivariate correlation of *r* ≈ 0.5 with 80% power at α = 0.05 typically requires around 30 participants), which may not have allowed us to detect the full extent of the network. This limitation might also explain why we observed only statistical trends between savings and two other pathways: one connecting the right cerebellar lobule VI to the ipsilateral ventrolateral thalamus, and another between the right cerebellar lobule VIIIb and the left supramarginal gyrus. Moreover, it is important to acknowledge that functional connectivity analyses generally have limited statistical sensitivity (Cisler et al. 2014; McLaren et al. 2012; Rissman et al. 2004). First, beta series connectivity relies on modeling individual beta coefficients for each trial, which substantially increases the number of regressors and thus reduces degrees of freedom for statistical testing. Second, this modeling produces relatively noisy beta estimates, lowering the signal-to-noise ratio. These two limitations are likely even more pronounced in our study since we focused on the parametric modulation by error of the main trial regressors. Consequently, some true connectivity pathways may have gone undetected due to this reduced statistical sensitivity.

Perhaps the most unexpected absence in our savings-related findings is that of the primary motor cortex. Numerous studies using non-invasive brain stimulation techniques have concluded that this region plays a central role in motor memory retention (Galea et al. 2011; Hadipour-Niktarash et al. 2007; Muellbacher et al. 2002; Orban De Xivry et al., 2011). However, this view has been challenged by studies that applied stimulation after the completion of training, rather than during, which may affect the learning process itself in ways that are difficult to measure and could indirectly impair retention. These studies reported preserved retention when stimulation was applied over the primary motor cortex, but impaired retention when applied over the primary somatosensory cortex (Baraduc et al. 2004; Darainy et al. 2023; Ebrahimi and Ostry 2024; Kumar et al. 2019). Although there is no definitive interpretation of these findings, one hypothesis is that the primary somatosensory cortex stores newly acquired sensory states that are later realized by motor areas. In this framework, the primary somatosensory cortex, known to receive actual sensory feedback, could plausibly encode sensory prediction errors. Our findings support this view, showing that its activity scales with movement error during adaptation and is also associated with motor memory retention as assessed by savings.

While our study, along with previous works (Debas et al. 2010; Mawase et al. 2017), supports the idea that savings rely on a cerebello-thalamo-cortical network, other brain regions have also been implicated, including the hippocampus and the default mode network (Cassady et al. 2018; Standage et al. 2023). Given the hippocampus’s established role in declarative memory, its involvement in savings has been interpreted as reflecting the recall of a cognitive or explicit component of visuomotor performance. In contrast, because the default mode network is typically anticorrelated with brain areas involved in cognitive control, its engagement has been taken to suggest the contribution of a more procedural or implicit component to savings. Regardless of the precise validity of these interpretations, it is reasonable to assume that multiple processes contribute to saving, a view aligned with the multiple-process account of human sensorimotor adaptation (Huberdeau et al. 2015). This framework posits two main learning mechanisms: an explicit process, tied to conscious strategies such as re-aiming, and an implicit process, which operates outside of awareness (McDougle et al. 2015; Taylor et al. 2014). Both processes can drive savings, with support for contributions from the explicit component (Avraham et al. 2021; Coltman et al., 2019; Morehead et al. 2015) as well as the implicit one (Coltman et al., 2019; Herzfeld et al. 2014; Leow et al. 2016; Yin and Wei 2020). Crucially, the explicit process can be approximated by the fast state of the state-space model, and the implicit process by the slow state (McDougle et al. 2015). In our study, only the learning rate of the slow component increased from day1 to day2, suggesting that the slow/implicit process was the main contributor to savings. This interpretation is further supported by the fact that the brain regions associated with savings in our data were motor, rather than cognitive, areas of the cerebellum, thalamus, and cerebral cortex. Supporting this distinction, Kim et al. (2015) demonstrated that the fast component engages prefrontal and inferior parietal regions, while the slow component is more closely tied to cerebellar structures, particularly lobule VI. Nonetheless, caution is needed in interpreting our findings as direct evidence for the slow component’s role in savings. Notably, we did not find correlations between connectivity and savings when savings was estimated using the two-state model but only with the one-state model, which conflates fast and slow processes. Definitively resolving this issue would require targeted experimental manipulations designed to isolate the contributions of each component (e.g., Huberdeau et al. 2015; Morehead & Orban De Xivry, 2021).

Finally, we explored whether any changes in resting-state functional connectivity occurred following the adaptation sessions, potentially overlapping with the connectivity pathways associated with savings. This investigation was motivated by a previous study that reported changes in resting-state brain connectivity after motor learning, particularly in regions involved in encoding errors during the learning task (Bernardi et al., 2018). This finding suggested that error-related activations extend into subsequent resting-state activity and contribute to motor memory formation. Although we observed some changes in resting-state connectivity after adaptation, we did not identify any intrinsic connections that were reinforced through repeated adaptation. Therefore, our data do not support the idea of brain plasticity persisting beyond the adaptation tasks to underpin savings. Future studies involving more intensive learning (e.g., additional movement trials or multiple sessions) that could amplify both behavioral and connectivity changes may be valuable in revisiting the issue of plasticity and in further exploring the relationship between intrinsic and task-evoked activity during motor adaptation.

In summary, we investigated the brain changes in individuals who exhibited savings while relearning a visuomotor rotation task. We observed increased functional connectivity between some subcortical (cerebellum, thalamus) and cortical (primary somatosensory cortex, anterior midcingulate cortex) sensorimotor regions involved in processing movement errors during relearning, which predicted the extent of savings. Thus, improved motor performance in individuals is likely due to enhanced communication between brain regions associated with error processing. This finding offers a concrete neural mechanism for the concept of increased error sensitivity, which has been proposed in previous studies on motor learning and savings. However, this improved connectivity did not persist in the resting brain after learning the motor task.

## Supplementary Information

Below is the link to the electronic supplementary material.

Supplementary Material 1

## Data Availability

Anonymized raw data and analysis/modeling scripts are stored on SUMMER, the data server of Grenoble-Alpes university, and are available from the corresponding author on reasonable request.
