# Supplementary material for: Savings in visuomotor learning are associated with connectivity changes within a cerebello-thalamo-cortical network encoding movement errors
Source: Brain Struct Funct. 2025 Oct 13;230(8):156. doi: 10.1007/s00429-025-03013-4 (PMC12518493; doi:10.1007/s00429-025-03013-4)
Supplement: Supplementary file 1 — Supplementary Material 1 [file 429_2025_3013_MOESM1_ESM.docx]

**SUPPLEMENTARY MATERIAL**


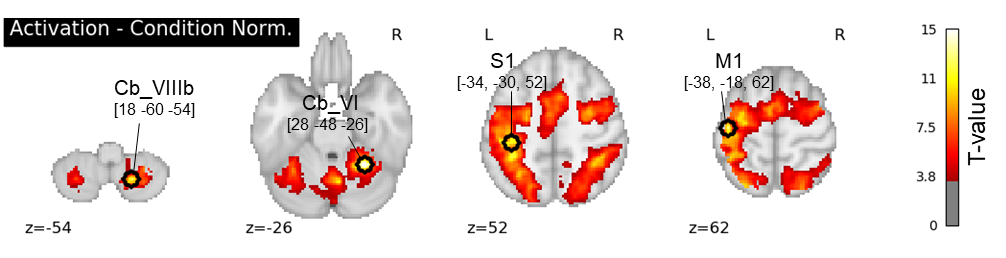


**Figure S1. ROIs locations.** ROIs have been defined from local maxima of the group-level activation map of the Norm condition (against implicit baseline).

M1: primary motor cortex; S1: primary somatosensory cortex; Cb_VI: cerebellar lobule VI; Cb_VIIIb: cerebellar lobule VIIIb; L: left hemisphere; R: right hemisphere.


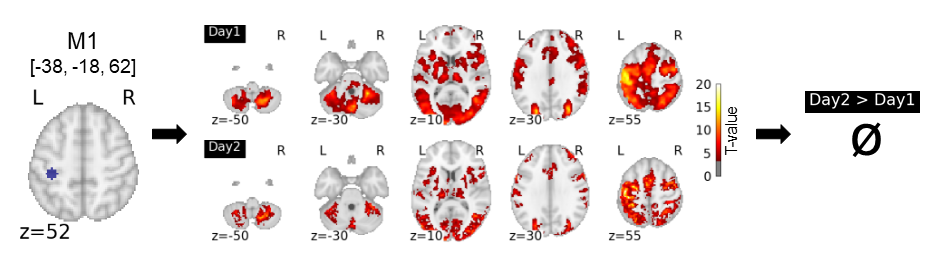


**Figure S2. Error-modulated M1-to-voxels co-activation patterns.** The contrast day2>day1 indicated that M1 connectivity related to error did not change on day2 compared to day1.


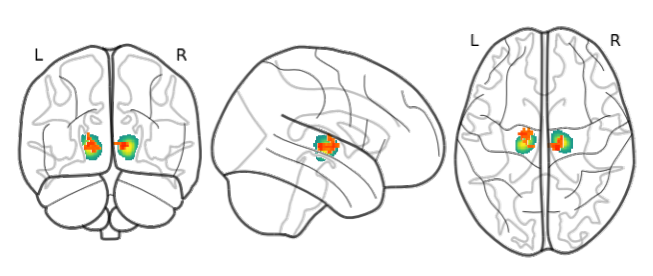


**Figure S3. Anatomical localization of cerebellar (lobule VI) error-modulated connectivity to the thalamus.** Stronger error-modulated connectivity observed during re-adaptation, illustrated by the hot (orange) map, overlaps with the ventral intermediate (VIM) nucleus, which is shown in the background as a cytoarchitectonic maximum probability map of Julich-Brain (Amunts et al., 2020). The Julich-Brain Cytoarchitectonic Atlas provides probabilistic representations of brain regions based on histological analysis of postmortem brains.

Amunts, K., Mohlberg, H., Bludau, S., & Zilles, K. (2020). Julich-Brain: A 3D probabilistic atlas of the human brain’s cytoarchitecture. Science, 369(6506), 988-992. https://doi.org/10.1126/science.abb4588
